# The Therapeutic Utility of Lactic Ferments

**Published:** 1909-12

**Authors:** 


### Abstracts and Selections.

### The Therapeutic Utility of Lactic Ferments.

What do we look for in a lactic ferment suitable for therapeutic application?

(1) A ferment which is able to run the gauntlet of the gastric juice, and establish itself within the intestine as a part of its flora.

(2) One that will give rise to lactic acid in the intestine in sufficient quantity to control to an appreciable extent the colon bacillus, and restrain undesirable fermentation in the ileum and colon.

Most pathogenic and saprophytic organisms multiply best in an alkaline medium, so that if the intestinal reaction can be rendered acid by lactic acid, we have an excellent means of inhibiting their proliferation in the intestine. This has been taken advantage of not only in the intestinal tract, but also in the vaginal, oral and nasal cavities.

Two of the most powerful lactic acid producers are the *streptobacillus* and *streptococcus lebinis* found in the Bulgarian milk ferments, and these have now been isolated and cultivated for several generations in the greatest purity in the Pasteur Institute of Paris. They seem to act well together and have the advantage of retaining their latent activities for a long time in the form of a tablet known as fermenlactyl.

Ordinary lactic ferments proliferate with difficulty when the temperature reaches 35 degrees to 36 degrees centigrade. At the temperature of 36 degrees centigrade, such lactic ferments as the *bacillus acidi paralactici, streptococcus lacticus,* etc., become enfeebled and have little antiputrifactive value in the digestive tube. These ferments, however, exist in fresh milk and good butter contains the *streptococcus lacticus,* but it is no longer active and is only able to exist with considerable difficulty in the gastro-intestinal tract on account of the body temperature.

The *bacillus subtilis,* which renders casein soluble, cannot develop to any extent in the presence of fermenlactyl, for after the acidity reaches a certain limit it arrests the action of the casein ferment.

The *streptococcus lebinis* multiplies more quickly than the *bacillus Bulgaricus* or *lebinis* at temperatures below 35 degrees centigrade, so that in fermenting milk with this ferment, when the temperature rises above this point, it is the *streptococcus lebinis* which predominates in the soured milk.

The *bacillus Bulgaricus* (or *streptobacillus lebinis*) and the *streptococcus lebinis* are therefore indispensable for the prevention of gastro-intestinal auto-intoxication since they produce as much as 1.5 per cent of lactic acid, while the *streptococcus lacticus* with difficulty yields even 1 per cent.

The conclusion to draw from this discussion is as follows: Fermenlactyl guarantees at all temperatures between twenty degrees and 55 degrees, the predominance of two species of lactic ferments which are really useful from a therapeutic standpoint.

Patients can be easily instructed how to make the buttermilk with fermenlactyl. While this requires some technical knowledge (when a pure culture is required), such as attention to complete sterilization of the milk and keeping a uniform temperature of 45 degrees centigrade for eight to twelve hours, this is unnecessary for all practical purposes. It suffices, therefore, to advise the patient to crush a tablet and add it to a quart of fresh, unsterilized milk (which thus retains the natural milk ferments), and to keep it in a warm place for twelve to fifteen hours in a corked wide-mouthed bottle. By this time a thick, very pleasant, nutty-flavored acid buttermilk is formed, teeming in useful lactic acid bacteria, while pathogenic and saprophytic bacteria are rapidly killed by the excess of lactic acid formed, leaving few, if any, living organisms besides the useful lactic ferments of fermenlactyl present. It should be remembered, however, that *equally good results are obtained by swallowing two tablets of fermenlactyl half an hour after each meal with a little milk or sugar and water.*

